# Adrenergic Signaling in Circadian Control of Immunity

**DOI:** 10.3389/fimmu.2020.01235

**Published:** 2020-06-23

**Authors:** Sarah Leach, Kazuhiro Suzuki

**Affiliations:** Laboratory of Immune Response Dynamics, Immunology Frontier Research Center, Osaka University, Osaka, Japan

**Keywords:** sympathetic nervous system, circadian rhythms, adrenergic, immunity, noradrenaline

## Abstract

Circadian rhythms govern a multitude of physiologic processes, both on a cell-intrinsic level and systemically, through the coordinated function of multi-organ biosystems. One such system—the adrenergic system—relies on the catecholamine neurotransmitters, adrenaline and noradrenaline, to carry out a range of biological functions. Production of these catecholamines is under dual regulation by both neural components of the sympathetic nervous system and hormonal mechanisms involving the hypothalamus-pituitary-adrenal axis. Importantly, both neural and hormonal arms receive input from the body's central clock, giving rise to the observed rhythmic variations in catecholamine levels in blood and peripheral tissues. Oscillations in catecholamine signals have the potential to influence various cellular targets expressing adrenergic receptors, including cells of the immune system. This review will focus on ways in which the body's central master clock regulates the adrenergic system to generate circadian rhythms in adrenaline and noradrenaline, and will summarize the existing literature linking circadian control of the adrenergic system to immunologic outcomes. A better understanding of the complex, multi-system pathways involved in the control of adrenergic signals may provide immunologists with new insight into mechanisms of immune regulation and precipitate the discovery of new therapeutics.

## Circadian Rhythms and the Adrenergic System

### Circadian Rhythms

Circadian rhythms are biological processes that exhibit intrinsic, self-sustained oscillations even in the absence of external, environmental cues. In mammals, these processes are maintained at the cellular level by transcriptional feedback loops that regulate expression of biological clock genes in a rhythmic fashion, following an approximately 24-h cycle. These cell-autonomous clocks are synchronized to function in concert at the bio-systems level, giving rise to the cyclic rhythms observed in, for instance, cardiovascular and endocrine function. Circadian rhythms are entrained by external signals (or “zeitgebers”) such as light/dark cycles, temperature, sleep and feeding patterns, which, although not required for the maintenance of endogenous oscillations, function to synchronize the biological clock with the surrounding environment. Circadian rhythms are controlled by a central, light-sensitive “master clock”—the suprachiasmatic nucleus (SCN)—that coordinates the function of peripheral, tissue-regulated- and cell-autonomous-clocks (1). This hierarchical organization supports the synchronization of multi-organ systems while simultaneously maintaining the ability to independently fine-tune or uncouple local responses to circadian signals.

The SCN is located in the hypothalamus directly above the optic chiasm where it receives input from photosensitive ganglion cells in the retina. Photic signals are transmitted by neural fibers from the retina along the retinohypothalamic tract (RHT), eventually initiating a neurotransmitter-driven signaling cascade involving glutamate, PACAP (pituitary adenylate cyclase-activating polypeptide) and aspartate (2, 3). The RHT terminates at the ventrolateral region of the SCN, forming direct contact with SCN neurons in this region (4, 5). Here, neurotransmitter signaling through NMDA (N-methyl-D-aspartate) or AMPA (α-amino-3-hydroxy-5-methyl-4-isoxazoleproprionic acid) receptors results in CREB binding to cAMP response elements in promoters of the so-called “clock genes” (6–9). Although this CREB-mediated regulation of clock gene expression is by far the most well-characterized mechanism linking circadian transcriptional feedback loops with photic signals, the search for alternative mechanisms is an area of active investigation (10). Thus, light cues are thought to be integrated into the molecular pathways governing circadian rhythmicity, providing a basis for photic entrainment of various biorhythms.

On a cellular level, control of circadian rhythmicity is thought to involve the interactions of at least two major autoregulatory transcription-translation feedback loops. The central players in the first feedback loop are the CLOCK (circadian locomotor output cycles kaput) and BMAL1 (brain and muscle aryl hydrocarbon receptor nuclear translocator-like 1) proteins (11, 12). CLOCK and BMAL1 interact to form a heterodimer which drives expression of PER (period circadian protein) and CRY (cryptochrome) proteins by binding to E-boxes in the *Per1, Per2, Per3, Cry1*, and *Cry2* promoters (13, 14). PER and CRY, conversely, act as transcriptional repressors by displacing CLOCK-BMAL1 from E-box regulatory elements (15). The second feedback loop involves the nuclear receptors REV-ERBα, REV-ERBβ, and RORα (retinoic acid receptor-related orphan receptor alpha) (16–18). REV-ERBα and REV-ERBβ themselves undergo cyclic, circadian expression under the transcriptional activation of CLOCK-BMAL1 and repression by CRY-PER, while also exhibiting repressive control of CLOCK and BMAL1 expression (18). RORα, on the other hand, competes with REV-ERBα to drive BMAL1 expression (19). Together, these interlocking, auto-regulatory transcription-translation loops constitute the molecular basis for the cyclic gene expression driving circadian biorhythms. More extensive reviews of the molecular mechanisms underlying circadian rhythmicity can be found elsewhere (20–22).

### The Adrenergic System

The adrenergic system is a neuro-hormonal system that regulates a range of physiological functions which are carried out through production of the catecholamines, adrenaline (epinephrine; EP) and noradrenaline (norepinephrine; NE). EP and NE signal through adrenergic receptors expressed on a wide variety of tissues and cell types, and are involved in processes such as regulation of cardiac function (23, 24), vascular remodeling and fat metabolism (25, 26), smooth-muscle-mediated vaso- and broncho-constriction (27), placental development (28), and control of immune function (29–31). Catecholamine production is regulated systemically via humoral messengers generated by the hypothalamus-pituitary-adrenal (HPA) axis, and locally by neural components of the sympathetic division of the autonomic nervous system. EP and NE are synthesized at a 4–1 ratio (favoring EP) (32) in the adrenal medulla and released into the bloodstream to carry out systemic functions. Neurons of the sympathetic nervous system (SNS), on the other hand, produce and predominantly secrete NE at discrete locations marked by the presence of adrenergic nerve terminals, thereby supplying peripheral tissues with highly localized NE signals. Importantly, the adrenergic system is one of the many biological systems thought to be under circadian control.

### Rhythmic Catecholamine Production

In 1943 Pincus (33) made the preliminary observation that the concentration of certain adrenal hormones in urine oscillated following a night-day pattern. Two decades later, isolated adrenal glands were found to exhibit intrinsic metabolic rhythmicity in culture, pointing to the existence of a self-sustained, endogenous clock (34). Following this discovery, a role for the SCN as a regulator of circadian adrenal function was suggested by ablation of circadian oscillations in adrenal corticosterone content following lesioning of the SCN (35). Consistent with these reports detailing both endogenous and exogenous control of circadian fluctuations in adrenal function, diurnal rhythms in plasma EP and NE levels were also described. Humans were found to have low circulating catecholamine levels during the night and high levels during the day (36), while rodents exhibited the opposite pattern (corresponding to opposite periods of activity) (37).

However, although EP and NE exhibited, overall, similar 24-h rhythms in circulation, many early studies reported differences in the maintenance of EP and NE oscillations under free-running conditions (or in the absence of entrainment). Specifically, EP was reported to exhibit clear, self-sustained rhythmicity, while NE levels were found to adjust rapidly to sleep/wake patterns, leading many to conclude that rhythmicity in circulating NE levels was just a result of sleep or even postural cues (36, 38–40). Later studies more clearly demonstrated this distinction by showing that NE cycles were abolished under constant light or food-deprivation conditions (40, 41). These findings led to the conclusion that while oscillations in circulating EP appear to be circadian and are regulated by the HPA axis, cyclic variations in circulating NE exist only in the presence of cyclic, external zeitgebers and, therefore, cannot be considered truly circadian according to the strictest definition.

There is evidence, however, that the release of NE from sympathetic neurons within tissues is under circadian control. This was pointed to, for example, by the finding that NE in cerebrospinal fluid (CSF) [which is likely neuron-derived, as NE does not readily pass the blood-CSF barrier (42)] exhibits a circadian rhythmicity that is maintained despite disruption in light cycles (43). In addition, NE turnover in the pineal gland was demonstrated to exhibit an endogenous, 24-h rhythmicity that was maintained in blinded rats. The authors concluded that NE turnover in the pineal gland likely reflected daily rhythms in NE release from sympathetic neurons within this tissue (44). As a related side note, neuron-derived NE signals activate pinealocytes in the pineal gland through β2-adrenergic receptors, driving circadian oscillations in the production of melatonin from its metabolic precursor, serotonin (44, 45). Melatonin has been demonstrated to exhibit a range of effects on immunity including control of cytokine production by neutrophils and lymphocytes (46, 47), inhibition of nitric oxide synthesis (48) and promotion of immune cell migration (49, 50), to name a few examples. Thus, circadian NE rhythms feed into rhythmic variations in indolamine metabolism, providing another, albeit indirect, way for the noradrenergic system to influence immune responses.

### Regulation of Systemic Catecholamine Production by the HPA Axis

Release of EP and NE into circulation has long been thought to be regulated by the hypothalamic-pituitary-adrenal (HPA) axis. Consistent with the observation that circulating catecholamine levels (particularly EP) exhibit circadian rhythmicity, neuroanatomical tracing studies demonstrated that SCN neurons of the circadian master clock are directly linked to the PVN (paraventricular nucleus neurons) of the hypothalamus—often considered the driver of the HPA axis (51). While the specific mechanisms by which signals governing circadian rhythmicity are propagated remain incompletely defined, various neurotransmitters have been proposed to be involved in SCN-PVN communication including vasopressin (52), vasoactive intestinal peptide (VIP) (53, 54) and neuromedin U (55). Moreover, the hypothalamus is known to release corticotropin-releasing hormone (CRH) and vasopressin in circadian fashion (52, 56). These PVN-derived peptides regulate pituitary function and drive the secretion of adrenocorticotropic hormone (ACTH), which then incites the adrenal cortex to produce glucocorticoids (cortisol in humans; corticosterone in rodents). Within the adrenal gland, glucocorticoids are transported to the medulla where chromaffin cells produce NE. Here, glucocorticoids activate the enzyme, phenylethanolamine *N*-methyltransferase (PNMT), which is required for the conversion of NE to EP (57, 58). Thus, cyclic variation in HPA signals are likely particularly important in driving diurnal oscillations in adrenal EP levels. Although our understanding of these processes remains far from complete, signals from the body's central, master clock are, in this way, thought to be translated into rhythmic variations in neurotransmitters and hormonal messengers, which influence peripheral sites in circadian fashion. This includes the cyclic release of catecholamines by the adrenal gland into the bloodstream.

### Regulation of Systemic Catecholamine Production by the Sympathetic Adrenal Medullary Axis (SAM)

In addition to HPA-mediated regulation (and of arguably greater physiological importance for the production of adrenal catecholamines), adrenal glands are also innervated by neurons connected (via a polysynaptic pathway) to the SCN, providing a mechanism for direct regulation of adrenal catecholamine production by the body's master clock (59). Innervation of the adrenal medulla by preganglionic sympathetic neurons emanating directly from the spinal cord (60, 61), as well as splanchnic (62) and vagus nerves (63, 64), has also been reported. Neural stimulation of chromaffin cells and subsequent catecholamine secretion is thought to be induced by cholinergic signals, much in the same way that post-ganglionic, sympathetic neurons are activated to secrete NE by pre-ganglionic neurons in sympathetic ganglia (65, 66).

**Figure 1:**
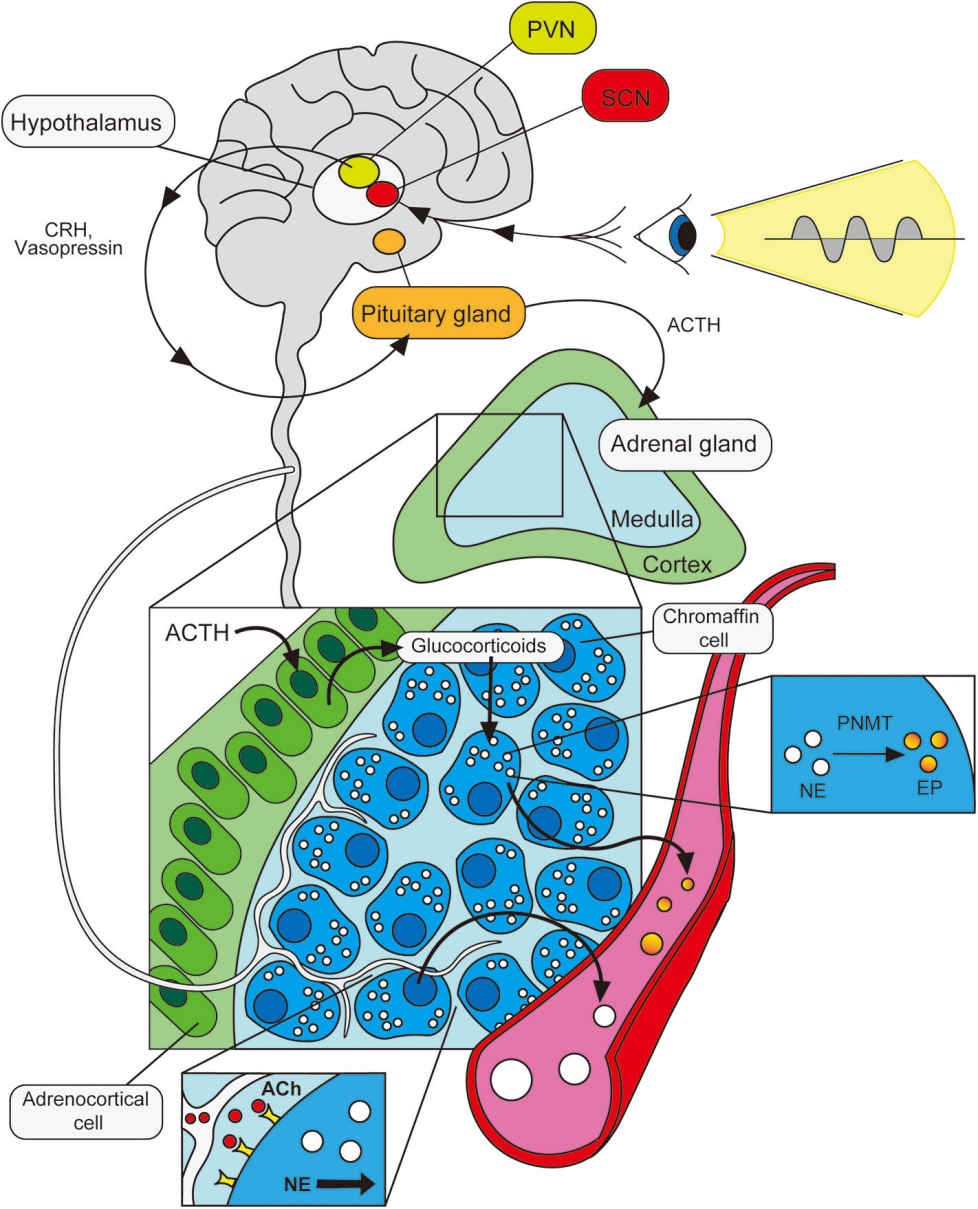
Circadian control of adrenal catecholamine output. Photic signals entrain the body's master clock in the hypothalamus (SCN), sending oscillating signals to the PVN. The PVN propagates these neural signals to the pituitary gland via various neurotransmitters and hormonal messengers. The pituitary gland responds by releasing ACTH, which drives adrenocortical cells in the adrenal gland to produce glucocorticoids. These signal to chromaffin cells in the medulla, activating the enzyme, PNMT, which catalyzes the conversion of NE stores into EP for release into the bloodstream. The adrenal medulla is also innervated by acetylcholine (ACh)-producing neurons which also respond to cyclic light signals in the SCN. Stimulation of these neurons activates chromaffin cells, driving the release of NE into the bloodstream in a cyclic fashion.

Consistent with these anatomical studies demonstrating neural connections between adrenal glands and the SCN, adrenal clock gene expression has been shown to be responsive to light entrainment (67). Furthermore, light-induced, acute activation of adrenal nerves is abolished in SCN-lesioned mice (68). This, combined with the observation that the rhythmicity in adrenal clock gene expression is unaltered in hypophysectomized rodents (69), lends support to the notion that adrenal glands may be subject to circadian oversight via neural, as well as hormonal, pathways. Thus, while HPA axis-mediated stimulation of chromaffin cells influences the ratio of EP to NE levels (70) in a cyclic fashion via glucocorticoid-driven PNMT activation (71), adrenal secretion of catecholamines also involves a local neural component—importantly, one that displays circadian rhythmicity (72) and is directly entrained by environmental cues organized in the SCN (68, 69). Although both the HPA axis and SAM axis potentially contribute to the oscillations in circulating catecholamine levels, the relative importance of these pathways under physiological conditions is not entirely clear. However, it has been suggested that the effects of the neuronal pathway may be manifest more prominently during prolonged or chronic disease states in which adrenal output and pituitary output do not correlate well (73–75) (Figure 1).

### Tissue-Localized Catecholamine Production by Sympathetic Neurons

Sympathetic neurons innervate, in addition to adrenal glands, a wide range of tissues including primary and secondary lymphoid organs (76, 77). The primary neurotransmitter thought to be produced by SNS nerve terminals is NE (78). In circulation, NE concentrations are reported to be in the picomolar to low nanomolar range (79), but under conditions of strong sympathetic stimulation, the tissue concentration at nerve endings may reach micromolar levels (80). Given the relatively low amount of NE in the blood, one might question whether circulating NE has a significant physiological role at steady state or under healthy conditions. Indeed, urinary concentrations of NE remain constant after adrenalectomy in humans (81) suggesting that neuron-derived NE in peripheral tissues may be more physiologically relevant for NE-specific functions than circulating NE. In regards to immune function, the observation that lymphoid tissue exhibits diurnal fluctuations in NE is particularly noteworthy, pointing to possible circadian control of SNS neuronal NE output (82).

### Adrenergic Receptors and Their Ligands

Catecholamines signal through adrenergic receptors, a class of G protein-coupled receptors. Broadly classified as α- or β-adrenergic receptors (α-ARs, β-ARs), α-ARs exhibit higher affinity for NE whereas β-ARs exhibit higher affinity for EP (83, 84) (although neither type of receptor exhibits exclusive binding to NE or EP). These two classes are further subdivided into three α1- (α_1A_, α_1B_ and α_1D_), three α2- (α_2A_, α_2B_ and α_2C_), and three β- (β1, β2 and β3) adrenergic receptor subtypes (85–88) which display varied tissue-tropism and activate distinct signaling pathways. While varying degrees of expression of all three AR types have been reported on different hematopoietic cells, β2-ARs are thought to be predominantly expressed (31) [albeit with a notable absence on both resting and activated type 2 helper T cells (89, 90)]. Examining expression of different receptor subtypes is hindered by lack of specific antibodies and receptor subtype-specific ligands. As a result, current understanding of receptor expression comes largely from transcript-level analyses or competitive binding/saturation studies; more comprehensive studies are still needed in this regard.

### Catecholamine Signaling Pathways

Signaling through adrenergic receptors reportedly modulates various cellular functions or properties, including (but not limited to) cell cycle and proliferation (91, 92), migration (93), and cytokine (94) and antibody (95) production. Although historically, signaling through β-ARs has often been associated with anti-inflammatory outcomes, in more recent years, the ability of β-ARs to mediate context-dependent, pro- and anti-inflammatory effects has become more widely appreciated (29, 94, 96). β-ARs signal through the G-protein, Gα_s_. Activation of Gα_s_ leads to activation of adenylate cyclase (AC), which catalyzes the conversion of adenosine triphosphate (ATP) into cyclic adenosine monophosphate (cAMP). Subsequently, cAMP activates protein kinase A (PKA), which is responsible for the activation of downstream transcription factors. Although all β-ARs signal through Gα_s_, it is thought that receptor subtype-specific differences in signal transduction are a result of specific localization of, for instance, membrane or cytosolic AC and PKA isoforms which form unique signalosomes, leading to the compartmentalization of cAMP pools which can then selectively target distinct physiological pathways (97–99). Non-canonical, G-protein-independent signaling through β-ARs has also been described (involving G protein-coupled kinases (GRKs) and β-arrestins), leading to the activation of ERK1/2 or MAPK pathways (100, 101). Intracellular cAMP levels have also been reported to influence, among other things, transcription of various cytokines in immune cells. For instance, increased cAMP leads to upregulation of IL-10 gene expression (102) while inhibiting IL-12 and TNF-α transcription (102, 103).

Much less is known regarding α-AR signaling on immune cells. Although not an exhaustive list, expression of α1-ARs has been reported on murine HSC progenitors (104), immature dendritic cells (93), mast cells (105), and human NK cells (106), while α2-AR expression has been reported on Kupffer cells (107), dendritic cells (108), NK cells (106), and occasionally on human lymphocytes (109, 110). Most of our current knowledge regarding signal transduction comes from studies of cardiomyocytes in which α1-ARs are coupled to Gα_q_ G-proteins. Ligand binding then leads to the activation of PLCβ1, downstream calcium signaling and activation of PKC (111). α2-ARs, on the other hand, associate with G_i_-type G proteins, and drive inhibition of AC and reduction of cAMP levels upon ligand binding (112, 113), potentially providing a means to counteract the effects of β-AR stimulation.

## Catecholamines in the Central Nervous System and Disease

### Catecholamine Production in the Central Nervous System

The locus coeruleus (LC) is the primary source of NE in the central nervous system, sending projections throughout the brain (114). It is thought to have a role in attention and arousal (115), as well as in stress-responses such as fear or anxiety (116, 117). Moreover, retrograde tracing experiments have revealed trans-synaptic circuits connecting the SCN and LC, and providing a basis for circadian control of LC activity (118, 119). In addition to circadian effects on LC firing, however, LC-derived NE levels both influence and are heavily influenced by sleep state (being completely absent during REM sleep) and changes in vigilance or alertness (120, 121), making rhythms in brain NE levels quite sensitive to fluctuation. This difficulty in assessing the specific circadian contribution to NE oscillations in brain tissue has proven a challenge to studies that might attempt to directly link circadian control of central catecholamine production and immune function. Nevertheless, indirect effects of LC-derived NE on immunity are expected to exist, for instance, through modulation of sleep and its general impact on whole body function.

### Catecholamines in the Central Nervous System and Immunity

The blood-brain barrier hinders direct interactions between LC-derived NE and most immune cell subsets. However, microglia—the brain's resident innate immune cells—are known to be highly sensitive to catecholamine signals, expressing higher levels of β2-adrenergic receptors than any other cell type in the brain (122, 123). Studies in mice have demonstrated an inhibitory role for LC-derived NE on microglial function involving changes in activation, nitric oxide production, tissue surveillance and gross morphology (124–126). Thus, one may easily imagine that circadian oscillations in NE coordinate microglia-neuron dynamics to promote homeostasis under normal physiological conditions, and that perturbations in adrenergic signaling might be particularly deleterious (or perhaps even a causative factor) in various neurological disease states. Indeed, LC dysfunction or degeneration is widely observed at early stages in both Alzheimer's disease and Parkinson's disease patients (127–129).

## Circadian Control of Immune Cell Trafficking Mediated by Adrenergic Signals

### Diurnal Variation in Leukocyte Trafficking Patterns

The most well-characterized means through which adrenergic signals exert circadian control over immunity is by regulation of cell trafficking. Diurnal variation in the number of blood circulating leukocytes was reported as early as the 1940s in both mice and humans (130, 131), with most reports describing peak blood leukocyte numbers during the inactive period. Some discrepancies exist, however, in the literature regarding cell-type-specific, day vs. night circulation patterns (130, 132–136). Nevertheless, this phenomenon seemed to be linked to adrenergic function as administration of adrenal or pituitary hormones resulted in blood lymphopenia (137) and adrenalectomy abolished cyclic variations in circulating leukocyte numbers (133, 138). As these early studies all pointed to a role for systemic (endocrine) catecholamine-mediated regulation of cyclic variation in leukocyte circulation, an important development in our understanding of this process was the finding that tissue-localized catecholamine signals originating from sympathetic nerve terminals could also significantly influence diurnal patterns in leukocyte migration between blood and peripheral tissues.

### Adrenergic Signaling in Stromal Cells Controls Immune Cell Egress From BM

Hematopoietic stem and progenitor cells (HSPCs) are constitutively released from BM into the circulation at steady state. However, mobilization of hematopoietic progenitors is dramatically increased under inflammatory conditions or “emergency” states in anticipation of increased requirements for hematopoietic output. Expression of CXCL12 within bone tissue is known to be important for retention of CXCR4^+^ HSPCs in hematopoietic niches and suppression of their mobilization. Conversely, granulocyte colony-stimulating factor (G-CSF) has long been recognized for its strong, mobilization-promoting properties. While exploring the mechanisms underlying G-CSF-induced HSPC mobilization, Katayama et al. made the somewhat surprising observation that destruction of peripheral adrenergic neurons by chemical sympathectomy (6-OHDA-(6-hydroxydopamine) administration), or treatment of mice with a β-AR antagonist led to a significant reduction in G-CSF-dependent, HSPC mobilization (139). This pointed to a role for neuron-derived adrenergic signals in this phenomenon. However, β2-AR agonist stimulation alone did not promote mobilization, suggesting that G-CSF and adrenergic signals somehow functioned cooperatively in this process. The authors also demonstrated a correlation between decreased osteoblast CXCL12 expression and HSPC mobilization. In conclusion they proposed that G-CSF suppresses CXCL12 production by osteoblasts, thereby promoting HSPC mobilization. In parallel, G-CSF and sympathetic neuron-derived NE signals were suggested to contribute to this process by some undefined mechanism. Although this initial study simply pointed to a role for adrenergic signals in HSPC mobilization, several subsequent studies have since sought to address the mechanistic questions raised by these observations.

**Figure 2:**
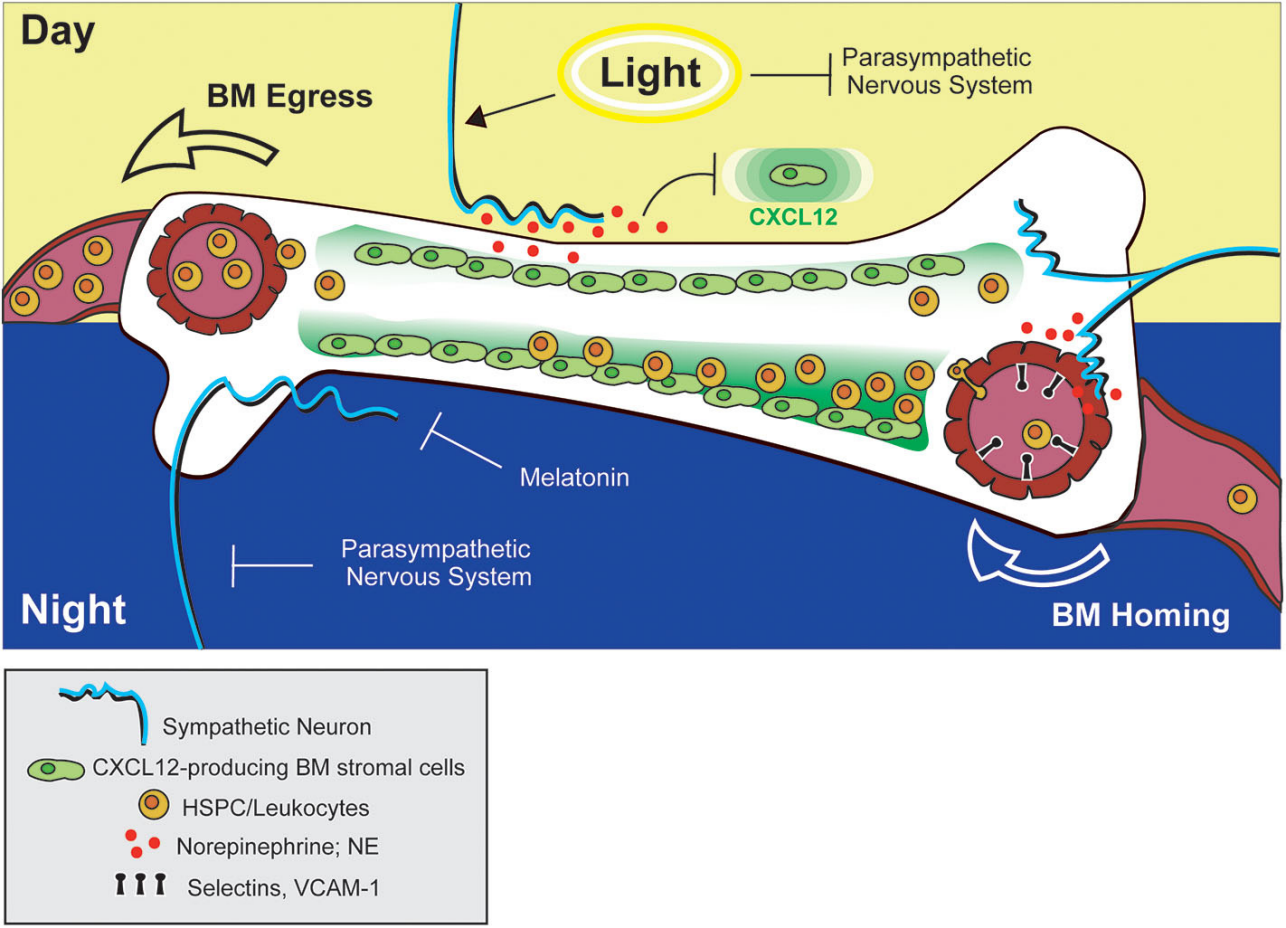
Adrenergic control of leukocyte and HSPC trafficking to and from BM. Circadian stimulation of sympathetic neurons drives down-regulation of CXCL12 in the BM, facilitating HSPC egress into circulation during the day (in mice). At night, adrenergic signals from sympathetic neurons promote upregulation of adhesion molecules by blood endothelial cells in bone tissue, promoting BM homing of leukocytes. The combined actions of melatonin and the parasympathetic nervous system may also function to prevent down-regulation of CXCL12 (and BM egress) during this time.

In 2008, Méndez-Ferrer et al. demonstrated that the diurnal variation in HSPC trafficking between BM and blood is a true, circadian phenomenon, as the observed rhythmicity was maintained under constant dark conditions and was abolished in *Bmal1*-deficient mice (140). The authors also confirmed, via surgical sympathectomy of the hind leg, that local, neuron-derived adrenergic signals in the BM were required for this process. Similar to the report by Katayama et. al., downregulation of CXCL12 expression was proposed to be the mechanism driving HSPC egress. However, in contrast to previous reports, Méndez-Ferrer and colleagues demonstrated that adrenergic signals were detected by β3-ARs (rather than β2-ARs) on some non-osteoblast, BM stromal subset [later proposed to be nestin^+^ mesenchymal stem cells (141)]. Moreover, stimulation of β-ARs with a non-subtype-specific β-agonist (isoprenaline) alone was sufficient to promote HSPC egress (Figure 2). This study provided intriguing evidence that circadian control of adrenergic signals may be discretely regulated in peripheral anatomic locations (such as the BM) by the sympathetic nervous system. The finding that localized, surgical sympathectomy could abolish circadian oscillations in HSPC output from BM, however, seems at odds with early studies reporting similar findings after adrenalectomy and raises questions regarding the relative importance of tissue-localized vs. systemic adrenergic signals in driving BM egress of immune cells. To our knowledge, a side-by-side comparison of the effects of adrenalectomy and surgical sympathectomy on circulating immune cell subsets has not been carried out, but would help shed some light on this issue.

### Adrenergic Signaling Mediates Immune Cell Recruitment to Peripheral Tissues

**Figure 3:**
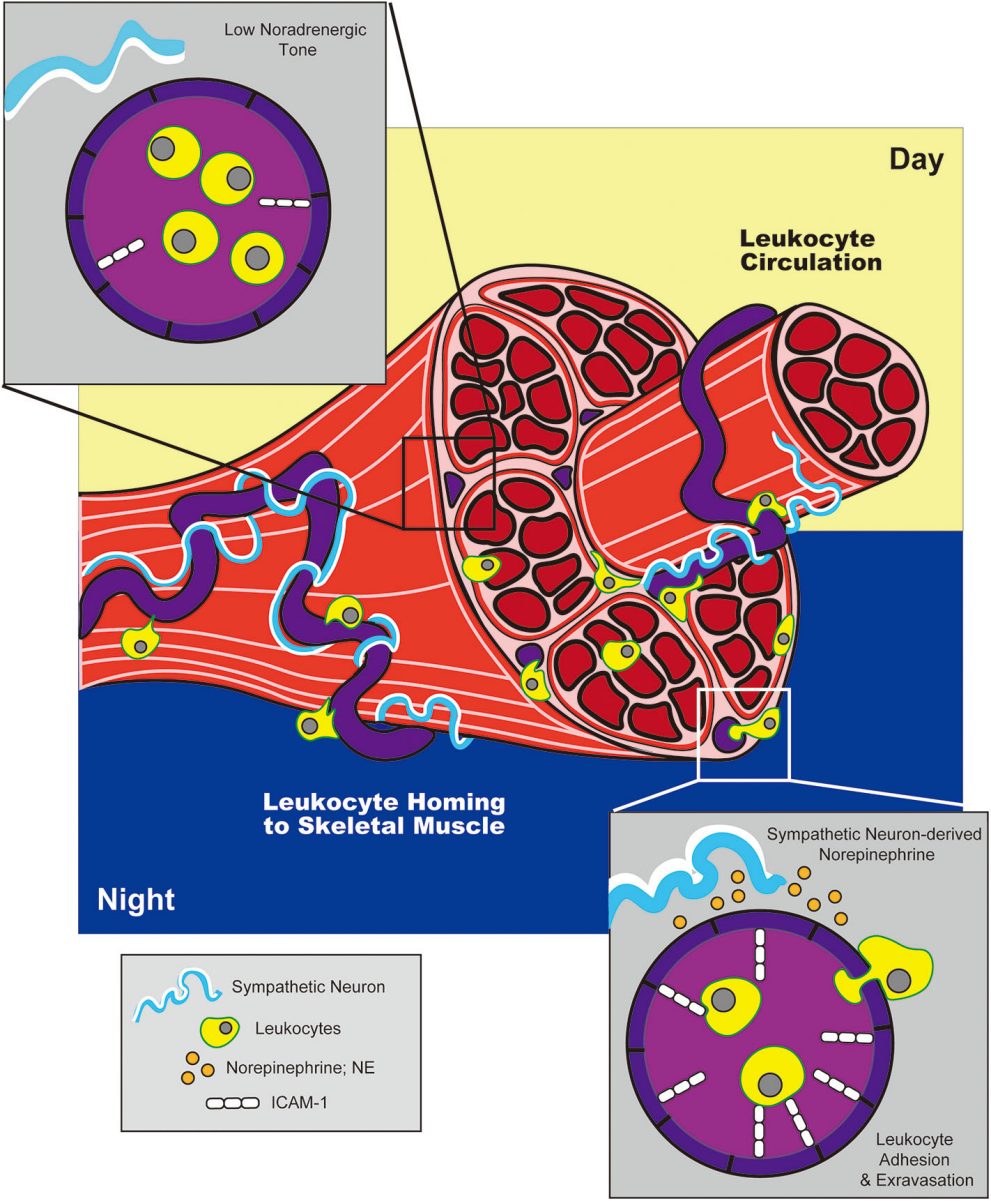
Adrenergic control of circadian leukocyte trafficking to skeletal muscle. During the night (in mice), adrenergic signals from sympathetic neurons drive upregulation of adhesion molecule, ICAM-1, on blood endothelial cells in skeletal muscle. This promotes leukocyte trafficking to and extravasation into tissue during the night, while reduced ICAM-1 expression during the day facilitates return to circulation.

The concept that adrenergic signals could influence circadian variations in HSPC circulation was later expanded by studies examining the effects of adrenergic signaling on immune cell trafficking to peripheral tissues at rest or under inflammatory conditions. Scheiermann et al. reported that variation in recruitment of leukocytes from blood to BM or skeletal muscle also exhibited diurnal tendencies, with rhythms oscillating in antiphase to that observed for HSPCs traveling from BM to blood (142). This phenomenon exhibited genuine, circadian characteristics as shown by ablation of oscillatory patterns in *Bmal1*-deficient mice (which lack a core component of the molecular circadian clock) and, conversely, maintenance of these patterns in the absence of light. The cyclic recruitment of leukocytes to peripheral tissues coincided with cyclic variations in expression of different tissue-specific adhesion molecules and chemokines expressed by endothelial cells at the site of extravasation. Diurnal variation in ICAM-1 and CCL2 was observed in skeletal muscle endothelial cells, while expression of P-selectin, E-selectin and VCAM-1 fluctuated within BM endothelium (Figures 2, 3). Importantly, surgical sympathectomy to denervate the BM or cremaster muscle abolished both cyclic leukocyte recruitment and oscillations in adhesion molecule expression, suggesting that, similar to their role in HSPC egress from BM, adrenergic nerves may provide tissue-specific signals that drive local changes in expression of guidance cues, thereby instructing immune cell trafficking in a diurnal fashion corresponding to the circadian nature of the adrenergic signals they provide. Adoptive transfer studies using receptor knock-out mice pointed to a role for both β2- and β3-ARs in this process (although only a requirement for β3-AR signaling could be confirmed using a similar agonist-based approach). Finally, the cyclic recruitment of leukocytes to peripheral tissues was shown to influence the pathological severity of various types of inflammatory challenge in disease models including septic shock and sickle cell vaso-occlusion (142).

One major question raised by these findings was how temporal changes in BM egress and recruitment could sustain overall cyclic patterns in immune cell circulation when both processes were reported to be governed by the same signals. While Scheiermann et al. reported that adrenergic nerves signal through β3-ARs on BM endothelium driving increased expression of selectins and VCAM-1 to promote immune cell recruitment to BM (142), Méndez-Ferrer et al. reported that adrenergic nerves signal through β3-ARs in BM to promote downregulation of CXCL12 expression and HSPC egress (140). How could these seemingly opposing mechanisms function in concert?

Related to this apparent paradox, Golan et al. reported that HSPC numbers in BM exhibit, not one but two, peaks and troughs during a single day/night cycle—one at night, and one after morning light exposure (135). The authors proposed that the morning HSPC peak coincides with a transient increase in NE and TNF levels in the BM driving β2-AR-mediated HSPC proliferation and differentiation, which is followed by a wave of HSPC egress into the bloodstream mediated by β3-AR-dependent processes. In addition, a second, night-time peak in BM HSPC numbers overlaps with a second NE and TNF burst during which melatonin was shown to inhibit HSPC egress and differentiation (Figure 2). In the context of the previously mentioned reports, these two different HSPC BM peaks could conceivably be attributed to the alternate adrenergic mechanisms driving BM egress and recruitment. Under this interpretation, the light-induced HSPC peak could represent the NE and TNF-induced HSPC proliferative burst that is followed by NE-driven egress via mechanisms described by Méndez-Ferrer et al. (140), while the night-time peak might represent the effect of BM recruitment driven by NE signals as reported by Scheiermann et al. (142). Although this dual peak model provided a potential explanation for altered temporal discernment of these two adrenergic-driven processes in mice, in humans, light exposure coincides with the beginning of the active phase. In this case, it would be expected that the light-induced and active-phase NE peaks would at least partially overlap, making it unclear whether such a model is equally relevant for both diurnal and nocturnal species.

Recently another layer of complexity was introduced to account for the different temporal responses to adrenergic signals in BM by studies examining the additional contribution of the parasympathetic nervous system and cholinergic signaling in this process. Using *Gfra2* (GDNF family receptor α2)-deficient mice as a model of parasympathetic deficiency, García-García et al. showed that parasympathetic nervous system-derived cholinergic signals suppress excessive sympathetic noradrenergic activity (143). At night, this may function to prevent β3-AR-mediated HSPC egress, while permitting β2-AR-driven BM homing. During the day, however, inhibition of parasympathetic activity by light exposure permits the egress of HSPCs from BM via noradrenergic, β3-AR-mediated mechanisms (Figure 2). Supporting this model, the authors also reported diurnal oscillations in β2- and β3-AR expression in BM, with β2-AR higher at night, and β3-AR preferentially expressed during the day (143).

### Immune Cell-Intrinsic Adrenergic Signaling

Initial studies examining the function of adrenergic signals in immune cell trafficking largely focused on the effects of catecholamines on non-hematopoietic cell types and their indirect effect on immune subsets. Nakai et al. brought further insight to the field with the finding that immune cell-intrinsic adrenergic signaling has the potential to alter a cell's migratory capability (144). This study began with the preliminary observation that treatment of mice with β2-AR agonists led to a rapid decrease in the number of circulating lymphocytes in both blood and lymph—a response that was largely lymphocyte-intrinsic, as later demonstrated using BM chimeras. Follow-up experiments showed that β2-AR stimulation effectively blocked lymphocyte egress from lymph nodes (LNs) leading to the concomitant reduction of lymphocyte numbers in circulation. Notably, while B cells, CD4^+^ and CD8^+^ T cells all responded to adrenergic stimulation, the effect was most pronounced in the B cell compartment, likely as a result of higher intrinsic β2-AR expression within this population. Moreover, LN-innervating sympathetic neurons were proposed to be the source of these adrenergic signals as 6-OHDA treatment led to a reduction in the number of lymphocytes retained in entry-blocked LNs (treated with integrin-neutralizing antibodies) compared to those in innervated controls (It should be noted, however, that the contribution from adrenal gland-derived catecholamines cannot be formally excluded). Prolonged β2-AR stimulation was shown to have a clear physiological impact on the severity of inflammatory T cell-driven responses in both the mouse model of multiple sclerosis, EAE, and delayed-type hypersensitivity (DTH) responses. These effects were mediated by the inhibition of T cell egress from LNs and, therefore, inhibition of migration to sites of disease progression (the central nervous system in EAE and the skin in DTH).

**Figure 4:**
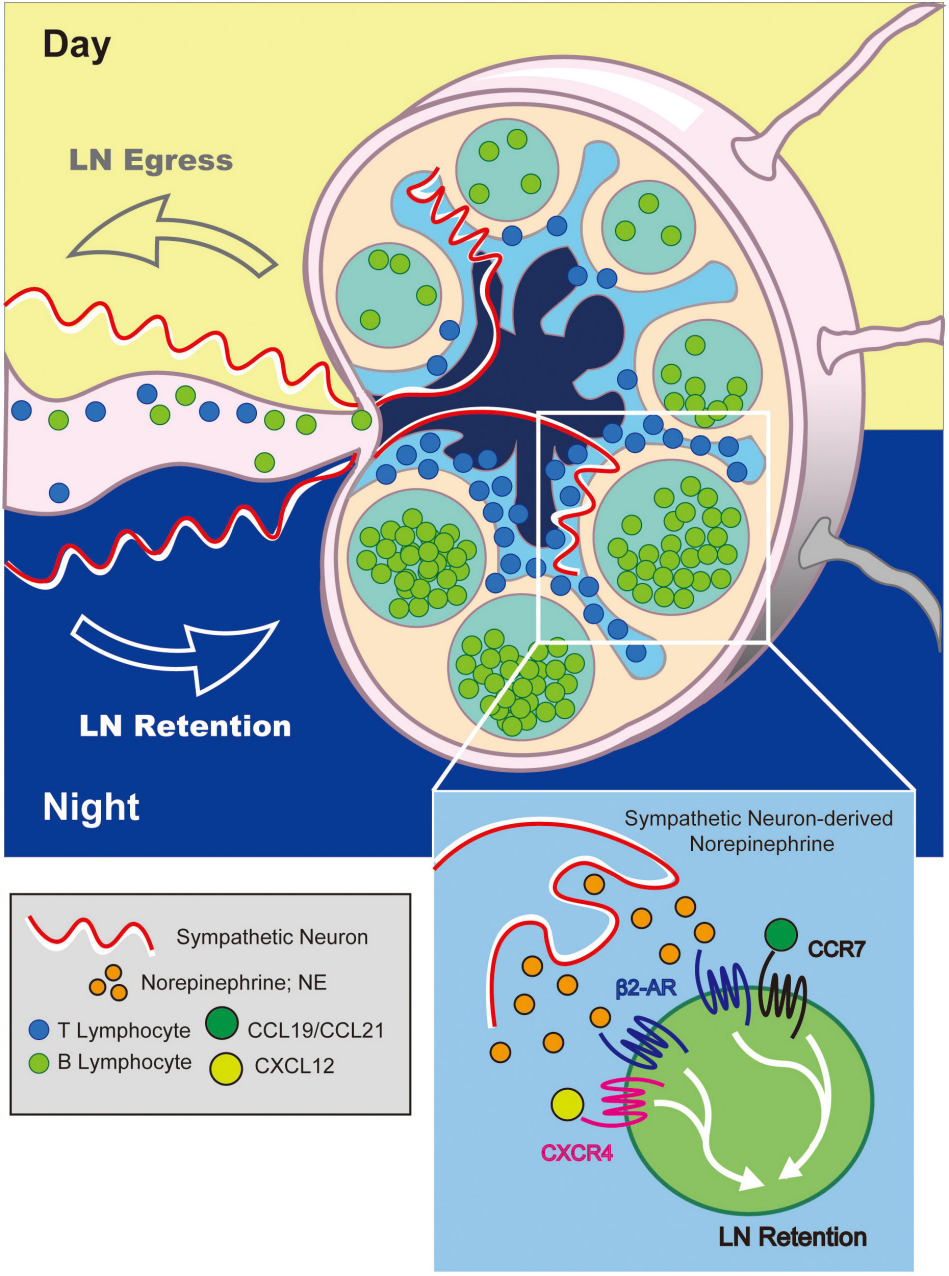
Adrenergic control of diurnal LN egress and lymphocyte retention. At night (in mice), adrenergic signals from sympathetic neurons stimulate β2-ARs expressed on LN-resident lymphocytes. This increases the sensitivity of lymphocytes to CCR7 and CXCR4-mediated LN-retention signals inhibiting their migration back into lymph fluid. Conversely, reduction in adrenergic tone during the day promotes lymphocyte egress.

The mechanism by which adrenergic signals promote LN retention of lymphocytes was shown to involve increased sensitivity of retention-promoting chemokine receptors, CXCR4 and CCR7, for their respective ligands following co-stimulation through the β2-AR (Figure 4). This was evidenced by prolonged Rac1 GTPase activation following co-stimulation, and was further supported by findings from membrane co-localization and immunoprecipitation experiments which pointed to potential physical interactions between β2-AR and CCR7 or CXCR4. Furthermore, *in vitro* migration assays demonstrated enhanced chemotactic responses in lymphocytes receiving dual β2-AR agonist and chemokine stimulation. Importantly, this mechanism was subsequently proposed to constitute the basis for diurnal oscillations in lymphocyte recirculation through lymph nodes in response to sympathetic neuron-derived signals (82).

Given that adrenergic signals are produced in a circadian manner from sympathetic nerve terminals, it followed that β2-AR-mediated control of lymphocyte retention in LNs might also contribute to the daily rhythmicity observed in circulating immune cells. In continuation of these previous studies (144), Suzuki et al. demonstrated that the diurnal fluctuations in blood- and lymph-circulating lymphocyte numbers varied in antiphase to that in LNs, with the active phase exhibiting high lymphocyte numbers in LNs, correlating with high LN adrenergic tone (82). These night/day fluctuations in LN lymphocyte numbers were abrogated after chemical denervation with 6-OHDA, suggesting that adrenergic nerves were providing the signals regulating cyclic LN egress. Moreover, the cyclic changes in lymphocyte numbers were dependent on lymphocyte-intrinsic β2-AR expression. Although not directly shown, the authors propose that this phenomenon likely also involved the enhanced sensitivity of lymphocytes to chemokine receptor stimulation under conditions of co-adrenergic stimulation as previously reported (144) (Figure 4). Finally, the authors demonstrated a very clear difference in the magnitude of the adaptive immune response elicited following immunization at night vs. during the day, with enhanced antibody, germinal center and T follicular helper cell generation evident after night-time immunization, when circadian NE signals in mice are known to be highest. Thus, this study clearly demonstrated that adrenergic signaling does not always lead to immuno-suppressive outcomes, but may, rather, support adaptive immunity to promote host defense in a context-dependent manner.

Apart from the reported role for adrenergic receptors in circadian lymphocyte trafficking through lymph nodes, others have reported that cell-intrinsic clocks may drive diurnal variation in lymphocyte numbers in secondary lymphoid organs (145). The role of cell-intrinsic clocks in lymphocyte function, however, has been somewhat disputed in recent years with some groups reporting no or limited effects (146) and others reporting, for instance, effects on lymphocyte differentiation (147). Given the multi-tiered organization of circadian control mechanisms, it may be argued that both central clock-mediated, adrenergic signaling and cell-intrinsic clocks may participate to an extent in this phenomenon. Although analysis of molecular, cell-intrinsic or central circadian clocks were not carried out in the study by Suzuki et al., sufficient evidence of circadian regulation of sympathetic neuron-derived NE signals exists to make a strong case for circadian control of lymphocyte egress from LNs as observed in this study (82).

In addition to these studies showing how lymphocyte-intrinsic adrenergic signaling may influence immune function, adrenergic signaling is likely to have varying cell-intrinsic effects on different hematopoietic subsets and is a topic of ongoing investigation. For instance, Spiegel et al. (148) reported that human CD34^+^ HSPCs upregulate expression of β-ARs in response to G-CSF treatment. The authors also provided evidence for synergistic effects of G-CSF and adrenergic signaling (by EP) in HSPCs, including enhanced proliferative and repopulation potential after engraftment. These effects were mediated, at least in part, by activation of the Wnt-β-catenin signaling pathway. The finding that G-CSF modulates immune cell responsiveness to adrenergic signals provided an alternative explanation for how G-CSF signals function in concert with neuron-derived adrenergic signals to promote HSPC mobilization as originally noted by Katayama et al. (139).

## Concluding Remarks

In this review we have attempted to give an overview of the ways in which the central circadian clock regulates rhythmic catecholamine synthesis and secretion, describe how catecholamines signal through adrenergic receptors in immune cell targets, and highlight a few publications which incorporate all of these aspects in a demonstration of the physiological significance of circadian regulation of immunity through adrenergic signaling. With this background regarding the ways in which circadian clocks regulate adrenergic signals in mind, the interested reader is encouraged to survey the many reviews detailing how adrenergic signaling more broadly affects immunity. We anticipate that future studies examining the effects of adrenergic signaling on immunity may be benefitted from a broader understanding of adrenergic signaling as a process regulated by circadian mechanisms, with potential implications for modulation of immune outcomes.

## Author Contributions

SL and KS both wrote and edited the manuscript. Both authors contributed to the article and approved the submitted version.

## Conflict of Interest

The authors declare that the research was conducted in the absence of any commercial or financial relationships that could be construed as a potential conflict of interest.
